# Low-Grade Central Osteosarcoma: A Difficult Condition to Diagnose

**DOI:** 10.1155/2012/764796

**Published:** 2012-07-16

**Authors:** A. M. Malhas, V. P. Sumathi, S. L. James, C. Menna, S. R. Carter, R. M. Tillman, L. Jeys, R. J. Grimer

**Affiliations:** The Royal Orthopaedic Hospital Oncology Service, Royal Orthopaedic Hospital, Bristol Road South, Birmingham B31 2AP, UK

## Abstract

Low-grade central osteosarcoma (LGCO) is a rare variant of osteosarcoma which is difficult to diagnose. If not treated appropriately, the tumour can recur with higher-grade disease. We reviewed our experience of this condition to try and identify factors that could improve both diagnosis and outcome. 18 patients out of 1540 osteosarcoma cases (over 25 years) had LGCO (1.2%). Only 11 patients (61%) were direct primary referrals. Almost 40% (7 of 18) cases were referred after treatment elsewhere when the diagnosis had not been made initially and all presented with local recurrence. Of the 11 who presented primarily, the first biopsy was diagnostic in only 6 (55%) cases. Of the remaining cases, up to three separate biopsies were required before a definitive diagnosis was made. Overall survivorship at 5 years was 90%. 17 patients were treated with limb salvage procedures, and one patient had an amputation. The diagnosis of LGCO remains challenging due to the relatively nonspecific radiological and histological findings. Since treatment of LGCO is so different to a benign lesion, accurate diagnosis is essential. Any difficult or nondiagnostic biopsies of solitary bone lesions should be referred to specialist tumour units for a second opinion.

## 1. Introduction

Osteosarcoma is the most common nonhaematological malignant primary bone tumour [1]. Low-grade central osteosarcoma (LGCO), or intraosseous well-differentiated osteosarcoma, is a rare intramedullary bone producing tumour [2]. It accounts for only 1-2% of all osteosarcomas and has an equal gender distribution [2, 3]. The majority of cases occur in the second and third decades [3].

The difficulty in the management of patients with LGCO is diagnosing the disease. Radiologically, the appearance of LGCO is often confused with that of fibrous dysplasia [4–8]. On histological examination, LGCO consists of fibroblastic stroma of variable cellularity and variable amounts of osteoid productions that are characteristically arranged in parallel seams resembling the pattern seen in parosteal osteosarcoma. The cytologic atypia is minimal, and occasional mitotic figures are always present [9]. There are some overlapping features with benign lesions such as fibrous dysplasia and desmoplastic fibroma [7, 9, 10]. The key feature to distinguish LGCO from the benign mimics is to identify permeative growth pattern [9]. Diagnosis is difficult on biopsy samples particularly when only the fibroblastic stroma devoid of cytologic atypia is represented and when permeation of the host trabecular bone cannot be demonstrated. LGCO can be confused radiologically with fibrous dysplasia, desmoplastic fibroma, nonossifying fibroma, osteoblastoma, and aneurysmal bone cysts [3–9, 11]. It is therefore vital to combine both radiological and histological findings to raise the suspicion of LGCO.

In a large case series of 80 patients from the Mayo clinic, the type of surgical management was found to strongly influence the prognosis [3]. Treatment by curettage or marginal excision was found to almost always result in local-recurrence. In those that recurred, 15% returned as a high-grade osteosarcoma with a worse subsequent prognosis. However, wide local resection with clear margins was found to have a much better prognosis [3]. The prognosis for LGCO has been reported to be over 90% at both 5 and 10 years [11, 12]. 

LGCO is therefore an eminently treatable malignant condition with a high chance of cure with appropriate treatment. If the diagnosis is not made in an accurate and timely fashion, then the prognosis is significantly worse, with a high chance of recurrence, often of higher-grade disease. The aim of this study was to review our experience of diagnosis and treatment of LGCO and highlight features that may lead to earlier diagnosis and, subsequently, better treatment and outcomes.

## 2. Methods

A retrospective review was performed of the orthopaedic oncology unit's database covering all patients referred from 1986 to the present (25 years). Inclusion criteria were a definitive histological diagnosis of LGCO at some stage in the patient's management. The identified cohort was then reviewed for age, gender, site, biopsy type, initial diagnosis, definitive diagnosis, treatment, outcome, and morbidity. All available radiographs, MRI, and bone scintigraphy were then reviewed by a dedicated musculoskeletal radiologist and the histological features reviewed by an experienced musculoskeletal pathologist. 

All needle and open biopsy samples were fixed in formalin and decalcified in 5% nitric acid as per local guidelines. The diagnosis of LGCOS was made on histological examination of haematoxylin and eosin-stained sections. Diagnosis was made on the presence of lesional tissue consisting of spindle cell proliferation, presence of cytologic atypia, presence of mitotic figures, and well-formed matrix production. Identification of permeative growth pattern was helpful in making a definitive diagnosis.

Survival time was analysed using the Kaplan-Meier survival method, and statistical significance was tested with the Mantel-Cox log-rank test.

## 3. Results

A total of 18 patients were identified with a definitive diagnosis of LGCO from a total of 1540 patients with osteosarcoma. LGCO therefore constituted a 1.2% subset of osteosarcoma. The gender distribution was equal with 9 males and 9 females and a mean age of 37 years at diagnosis (range 11 to 72 years) (Table 1). 

The majority of the tumours occurred in the lower limb (15 cases: 10 femur, 4 tibia, 1 os calcis). There were two cases in the pelvis and one in the upper limb. The most common presentation was that of pain in the affected limb, usually described more as a background ache than as a severe pain. The average duration of symptoms prior to presentation was over 2 years (range 4 weeks to 10 years). 

A review of the available radiographic imaging studies revealed that the mean length of the tumour at presentation was 9.9 cm (range 8–14). The appearance was predominantly mixed, sclerotic, and lytic, with well-defined margins. Two lesions were found to be lytic lesions with internal trabeculations. The majority of lesions were central although 3 were subarticular and 1 lesion was diaphyseal (final lesion was in the pelvis). Seven of the cases demonstrated a clear cortical breach, 2 were expansile, and only two lesions were contained. All but three demonstrated extraosseous masses on radiographs. 

On review of the MRI investigations, the tumour predominantly showed intermediate signal on T1 with one case being of low signal. All cases demonstrated an extraosseous mass and cortical disruption on MRI. Cystic areas were present centrally within the tumour in 4 cases with a single tumour showing fluid levels. All of the bone scans available for review demonstrated that the lesions gave a “hot” signal.

Eleven of the patients were referred directly to the unit for full diagnostic evaluation. The remaining 7 patients were investigated and treated elsewhere prior to referral.

Of the 11 patients referred directly to the unit, a primary diagnosis of LGCO was made in only 6 of the cases at the initial biopsy (3 open biopsy and 3 needle biopsy). A further 5 patients required multiple biopsies (2-3) to make the correct diagnosis. Three patients required 3 or more biopsies before the final diagnosis was made. In all three cases, the initial samples lacked cytologic atypia and a permeative pattern and the lesions appeared benign. In one case, the definitive diagnosis was made on the final resection sample. In the other two cases, the radiological studies were highly suggestive of a malignant lesion and so only after repeated sampling did the histology demonstrate enough cell atypia and infiltration to confirm a diagnosis of LGCO.

In one case, a diagnosis of fibrous dysplasia was given by a nonspecialist histopathologist and the patient was referred to the unit for curettage. The curetted material then gave a diagnosis of LGCO. In another case, an initial biopsy suggested atypical fibrous dysplasia which was treated with bisphosphonates. When the pain did not improve, he underwent curettage and again the histology showed atypical fibrous dysplasia. The lesion healed and the pain resolved, but within 15 months the pain recurred and he underwent a further curettage with a fibula strut graft and DHS fixation of the femoral neck. Histology, again, did not give a diagnosis of LGCO but rather fibrous dysplasia and osteofibrous dysplasia. The pain never resolved after this operation and gradually the fibula graft was resorbed. At that stage, a proximal femoral replacement was carried, removing all of the previous abnormal area. Analysis of the whole specimen confirmed the diagnosis of LGCO but with some areas now showing progression to intermediate grade. 

**Figure 1:**
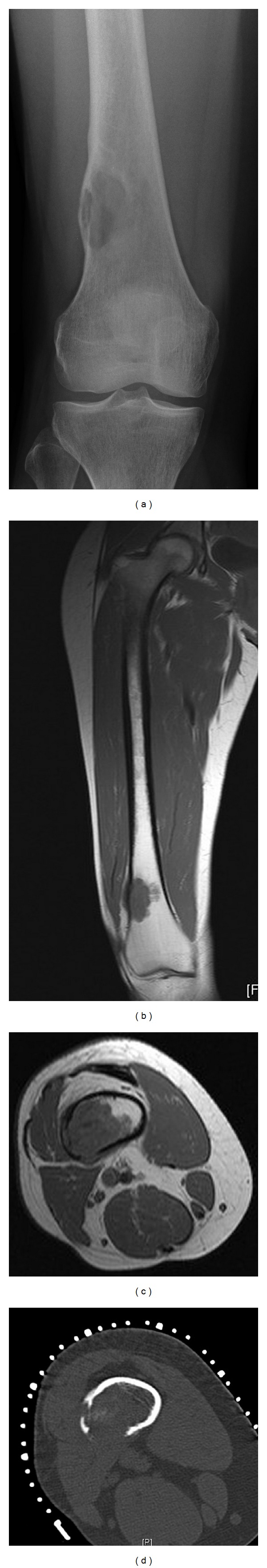
Radiological appearance of LGCO: a patient presented with a solitary bone lesion (a). MRI demonstrated an intermediate signal intraosseous lesion relative to skeletal muscle on T1 (b) and (c). CT-guided biopsy was undertaken (d). The lesion had marked cortical involvement with extraosseous expansion.

Of the 7 patients who were initially treated elsewhere, all had an initial diagnosis other than LGCO. These included fibrous lesion (benign and malignant), giant cell tumour, and simple bone cyst. Seven patients had undergone prior curettage (with or without bone graft or cement), and one had a local excision (see Figure 1 for an example). All of these patients presented with local recurrence, with two cases revealing a higher-grade osteosarcoma, but review of the original histology confirmed the retrospective diagnosis of LGCO. 

Four patients sustained a pathological fracture. Only one occurred prior to biopsy, the rest occurred prior to definitive surgery. 

For definitive treatment, 14 patients underwent wide local excision and endoprosthetic replacement, 3 patients underwent wide local excision and fibular grafting, and one patient underwent an amputation. The patient requiring an amputation was referred for initial investigation of a calcaneal lesion and only required one biopsy.

A total of seven patients received chemotherapy. Of the seven, four had chemotherapy when they presented with high-grade recurrence and three patients received it as they were thought to have higher-grade tumours but had the diagnosis revised to LGCO following the histology of the resection specimen. 

Of the 7 patients who presented with local recurrence, two had high grade tumours (one probably radiation induced). All of these patients remain alive and well following further treatment.

**Figure 2:**
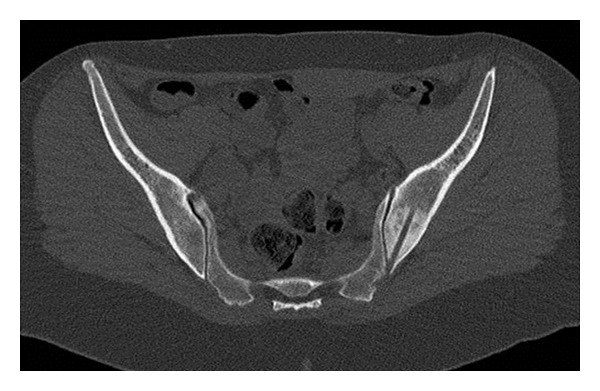
CT scan of the pelvis of a 33-year-old lady with low back pain. X-rays were normal as was an MRI of the lumbar spine. A bone scan showed increased activity in the ilium adjacent to the sacroiliac joint, and a CT showed a dense sclerotic lesion. This would have been typical for fibrous dysplasia radiologically, but this did not explain the pain. CT-guided needle biopsy confirmed the diagnosis. This CT image clearly shows the lesion and the path of the biopsy.

Of the 9 patients treated primarily at our unit, there were two recurrences, both as higher-grade tumours. One patient developed local recurrence and lung metastases 102 months following wide resection and a tibial endoprosthetic replacement. Histology confirmed high-grade recurrence, and the patient had palliative treatment, surviving for 7 months. The second patient presented with a pelvic lesion after 18 months of hip pain. Two CT-guided biopsies were inconclusive, suggestive of fibrous dysplasia or a low-grade sarcoma (Figure 2). Surgery was undertaken in the form of an en bloc excision of the ilium and reconstruction with fibula grafts. The specimen confirmed a 15 × 11 cm LGCO. The patient made a good recovery. At 15 months, two small nodules were noted in the scar. Excision revealed a locally recurrent high-grade osteosarcoma. Despite chemotherapy and a hindquarter amputation (giving clear margins with 60% necrosis), the patient developed lung metastasis and further local recurrence at 1 year. The patient was then treated with palliative radiotherapy for symptomatic relief.

As mentioned above, a total of four of the nine patients who developed recurrence of the tumour had reoccurred with a higher grade to tumour (44%) and three of the nine patients developed metastases (33%).

Disease-free survival for all patients was found to be over 90% at 5 years and 80% at 10 years. When the survival rates were compared between patients referred directly and those treated elsewhere, the survival curves were not statistically significant.

## 4. Discussion

This study demonstrates that several biopsies may be required to make the diagnosis of LGCO, even in a primary referral. This case series demonstrates that only 6 of the 11 patients treated primarily had a clear diagnosis of LGCO after the first biopsy. Seven patients presented with local recurrence of a LGCO that was not initially identified. Even in a specialist tumour centre, biopsy yielded an initial diagnostic rate of only 55% (6 out of 12). Five patients required multiple biopsies before the definitive diagnosis was made. The literature is in agreement with our findings, demonstrating the difficulty in recognising such a rare variant of osteosarcoma [11, 12]. Any unit seeing and treating patients with bone abnormalities must be aware of this potential diagnosis and have a high level of suspicion in any case where the clinical appearances, the radiology, and the histology do not match.

The classical case is therefore a patient in the 2nd or 3rd decade with a long history of mild-to-moderate pain who is found to have a fibroosseous lesion, frequently with a cystic component and in whom the histology is similar to but not typical of fibrous dysplasia. 

Larger biopsies and second opinions may then clarify the diagnosis and ensure that appropriate treatment is carried out. 

The radiological features are variable, as reported in the literature and confirmed in this series, demonstrating a mixed lytic and blastic appearance. Andresen et al. [13] described four radiographic patterns of low-grade central osteosarcomas (in 70 cases): (1) lytic with varying amounts of thick and coarse trabeculation (31%); (2) predominantly lytic with few thin, incomplete trabecula (30%); (3) densely sclerotic (24%); (4) mixed lytic and sclerotic (14%) [14]. By contrast, in this series, the majority of radiographic patterns were in keeping with mixed lytic and sclerotic lesions (80% type 4). Only two cases were found to be lytic with trabecular patterning (type 1 and 2). 

**Figure 3:**
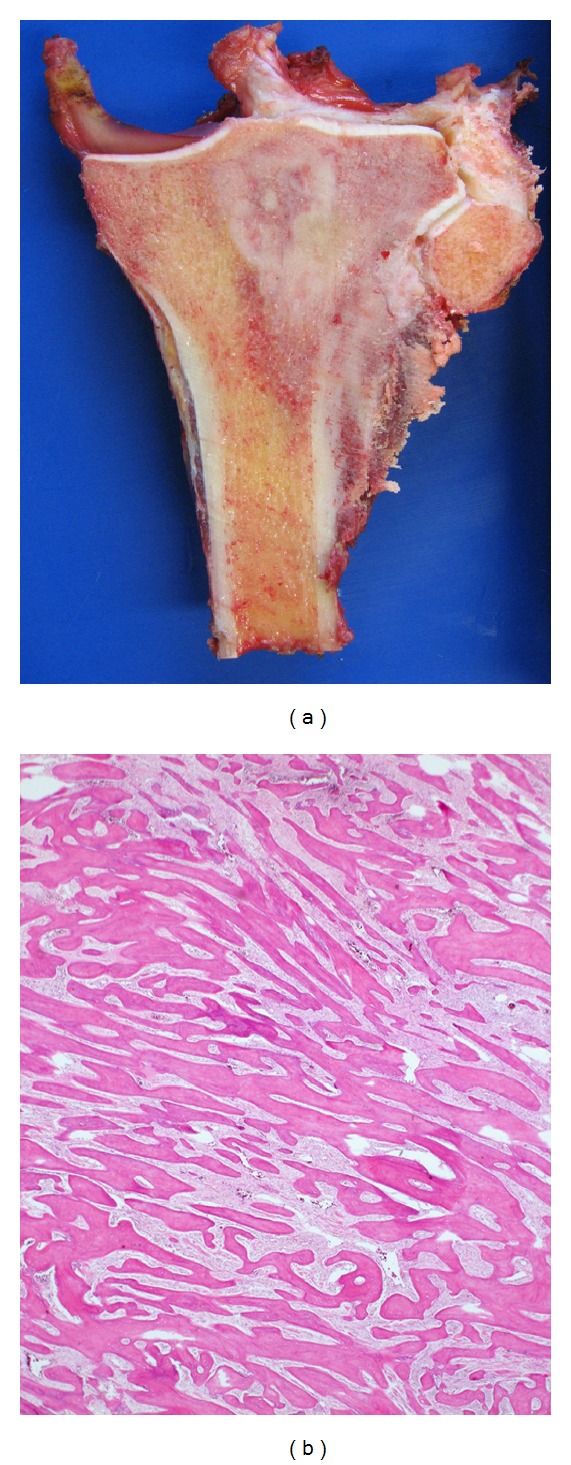
A histological example of LGCO: macroscopically, this greyish white tumour shows extramedullary extension (a). Microscopic review (haematoxylin and eosin stain, ×10) demonstrates (b): low-grade intramedullary osteosarcoma, which consists of parallel seams surrounded by spindle cell stroma which exhibits very minimal cytological atypia. The appearances are similar to parosteal osteosarcoma or fibrous dysplasia, for which it can be mistaken.

Histologically, LGCO is a malignant intramedullary bone forming neoplasm in which the tumour is so well differentiated that it is difficult to make a diagnosis of malignancy on limited biopsy samples [7, 10] (see Figure 3). These tumours are usually well-demarcated firm whitish masses that have a fibrous whorled appearance, typically centred in the medullary cavity of metaphysis or meta-diaphyseal region. Microscopically, the tumours are hypo-cellular consisting of interlacing fascicles of spindle cells. These spindle cells show mild cytological atypia, and mitotic activity is low. The tumour typically has a permeative growth pattern entrapping native bony trabeculae. There may be an associated soft tissue mass. The matrix produced is variable and well differentiated. On a biopsy material, LGCOS can be indistinguishable from benign fibrous lesions such as desmoplastic fibroma and fibrous dysplasia. LGCO is distinguished from benign tumours by virtue of its infiltrative growth pattern. Desmoplastic fibroma lack matrix production and fibrous dysplasia does not exhibit a permeative growth pattern. Histologically LGCO has morphological similarities with parosteal osteosarcoma which is a surface tumour. Therefore, close clinical radiological correlation is necessary in distinguishing these entities. 

Immunohistochemical stains murine double-minute type 2 (MDM2) and cyclin-dependent kinase 4 (CDK4) are presenting viable solutions to aid the histological diagnosis of LGCO. Because these patients were treated prior to the availability of these recent advances, none of the patients in this study underwent these immunohistochemical tests. Cyclin-dependent kinase 4 (CDK4) and murine double-minute type 2 (MDM2) genes (found on chromosome 12 q13–15) are genetic markers with a reported sensitivity of 89–100% and a specificity of 97–100% (when either one or both genes are present) in various studies [15, 16]. However, given the rarity of LGCO [2, 3], the clinical, radiological, and histological picture is still vital in raising the suspicion of LGCO in order to apply these tests.

The imaging appearance of LGCO can often be indistinguishable from fibrous dysplasia or parosteal osteosarcoma [3, 9, 17]. Even in the setting of an experienced multidisciplinary meeting, the diagnosis is often difficult to make but should be considered if atypical “fibrous dysplasia” is encountered.

It is generally accepted that management is with surgical excision with wide margins and that chemotherapy and radiotherapy are not routinely required. The main risk is from local recurrence which may be delayed (5 to 10 years or more) and is related to inadequate excision margins [3, 11, 12]. The literature suggests that 15% of recurrences will be high grade [3], but in this series four of the nine patients with recurrence had higher grade tumours (44%) and three of these four patients developed metastases, although only one patient has died thus far. 

Although this series contains only 18 patients, the sample size reflects the rarity of the disease. Patients referred secondarily to our centre may also suffer from a selection bias for inclusion. Despite these limitations, the study does demonstrate clearly the difficulty in arriving at the correct diagnosis and it also demonstrates the relatively good long-term prognosis when treated appropriately. When compared to other survivorship series within the literature, the study size is comparable. The largest historical series was published by Kurt et al. [3] over 20 years ago. Although it encompassed 80 cases, only 15 of those patients had follow-up data. The largest survivorship series was published by Choong et al. [11] in 1996 reporting on 20 patients. There are many smaller case reports within the literature dealing with a handful of patients [4–8].

This study has highlighted the problems previously reported by others of difficulty in obtaining the diagnosis and has confirmed that wide excision and limb salvage in most cases have a high chance of cure. We strongly recommend that any bone lesion proving difficult to diagnose should be referred to a specialist centre for evaluation. If a needle biopsy is not diagnostic, then a generous open biopsy has been found to be invaluable. It is important to take part of the lesion and, most importantly, a part of the margin with the adjacent, normal bone to offer the best chance of obtaining a diagnosis. As demonstrated, even a negative biopsy result should be treated with caution and referred to a tertiary sarcoma centre (with an experienced specialist pathologist). Once diagnosed, treatment is relatively easy with complete excision. The prognosis once treated appropriately is excellent.

## Figures and Tables

**Table 1:** Patients within the case series.

Gender	Age at diagnosis	Site	Primary or secondary referral	Previous diagnosis	Previous treatment	Definitive treatment	Chemotherapy	Followup (months)	Outcome
M	11	Mid tibia	Secondary	Osteoma	Excision “osteoma,” recurred after 1 year	Resection and fibula graft		186	No evident disease
F	14	Distal femur	Primary		None	EPR	Thought to be high grade	48	No evident disease
M	18	Distal femur	Secondary	Mesenchymoma	Curettage, LR after 4 yrs, recuretted, further LR 3yrs	EPR	90	No evident disease
M	19	Distal femur	Secondary	Cyst	Curettage and bone graft of fracture through cyst, LR 9 months	EPR	170	No evident disease
F	19	Distal femur	Secondary	Desmoplastic fibroma	Curettage and fixation of fracture through cyst, LR 3 yrs, recuretted and bone grafted, slowly progressive lesion for 4 years	EPR	24	No evident disease
M	29	Proximal femur	Primary	Atypical fibrous dysplasia	Curettage, then curettage and bone graft	EPR	As higher grade on LR	4	No evident disease
M	32	Distal femur	Secondary	Cyst	Curettage of cyst, uncertain diagnosis, post op RT, recurrence after 7 yrs as high grade tumour, lung met	EPR and thoracotomy	As higher grade on LR	7	No evident disease
F	33	Ilium	Primary		None	Excision		10	No evident disease
M	37	Proximal humerus	Secondary	Giant cell tumour	Curettage, LR 9 months with high-grade areas	EPR	As higher grade on LR	212	No evident disease
F	38	Ilium	Primary	Atypical fibrous dysplasia	Biopsy x3	Resection and fibula graft	As higher grade on LR	57	LR 14 months, high grade, chemo and amputation, alive with metastases
F	43	Distal femur	Primary		None	EPR		64	No evident disease
F	45	Proximal tibia	Primary	MFH	None	EPR	As initially thought be MFH	109	LR and metastatic disease at 102 months died 109 months
F	47	Distal femur	Primary	Fibroosseous lesion	Biopsy x2, curettage and cementation	EPR	Thought to be high grade after curettage	16	No evident disease
M	48	Calcaneum	Primary		None	Amputation	60	No evident disease
M	51	Distal femur	Primary		None	EPR	156	No evident disease
F	54	Distal femur	Primary		None	EPR	171	No evident disease
M	55	Proximal tibia	Primary	Fibrous dysplasia	Biopsy suggested fibrous dysplasia, curettage led to diagnosis	EPR	144	No evident disease
F	72	Proximal tibia	Secondary	Cyst? malignant	Curettage and bone graft of cyst, recurred 40 yrs later as LGCO	EPR	52	No evident disease

Key: RT: radiotherapy, CT: chemotherapy, LR: local recurrence, EPR: endoprosthesis, MFH: malignant fibrous histiocytoma, LGCO: low-grade central osteosarcoma.
